# Development of a Low-Cost Distributed Computing Pipeline for High-Throughput Cotton Phenotyping

**DOI:** 10.3390/s24030970

**Published:** 2024-02-02

**Authors:** Vaishnavi Thesma, Glen C. Rains, Javad Mohammadpour Velni

**Affiliations:** 1 School of Electrical and Computer Engineering, University of Georgia, Athens, GA 30602, USA; vaishnavi.thesma25@uga.edu; 2 Department of Entomology, University of Georgia Tifton Campus, Tifton, GA 31793, USA; grains@uga.edu; 3 Department of Mechanical Engineering, Clemson University, Clemson, SC 29634, USA

**Keywords:** big data, distributed computing, cotton phenotyping, computer vision

## Abstract

In this paper, we present the development of a low-cost distributed computing pipeline for cotton plant phenotyping using Raspberry Pi, Hadoop, and deep learning. Specifically, we use a cluster of several Raspberry Pis in a primary-replica distributed architecture using the Apache Hadoop ecosystem and a pre-trained Tiny-YOLOv4 model for cotton bloom detection from our past work. We feed cotton image data collected from a research field in Tifton, GA, into our cluster’s distributed file system for robust file access and distributed, parallel processing. We then submit job requests to our cluster from our client to process cotton image data in a distributed and parallel fashion, from pre-processing to bloom detection and spatio-temporal map creation. Additionally, we present a comparison of our four-node cluster performance with centralized, one-, two-, and three-node clusters. This work is the first to develop a distributed computing pipeline for high-throughput cotton phenotyping in field-based agriculture.

## 1. Introduction

The agriculture sector is currently challenged to produce sufficient food, crops, and fiber for the rapidly growing world population. Specifically, there is need for farmers to increase farm yield by at least 70% to meet the rising demand, especially for grain row crops, including wheat, rice, corn, and soybean [1,2,3]. However, the pressing challenges include water scarcity, a lack of arable land, reliance on manual labor, crop disease prevalence, and a lack of skilled workforce [4]. These prevailing challenges have motivated researchers to use modern tools such as unmanned aerial and ground vehicles with on-board processors and computer vision methods to help farmers monitor crops for field status and yield prediction for the past two decades [5].

The recent affordability and vast availability of computational boards, cameras, and storage devices in the past two decades have resulted in massive amounts of data being generated and stored from farms and controlled environments [6,7,8]. However, traditional processing methods cannot adequately process vast amounts of data in a timely and organized manner [9]. Furthermore, systematic processing of vast amounts of data requires extensive expertise in the domain of interest as well as data processing methods [4]. Thus, it is necessary to use big data tools to manage and automatically process the collected data in near real-time for plant phenotyping and crop monitoring [1,7].

In particular, cotton farmers experience challenges in yield prediction as the number of cotton bolls and their locations determine yield count and profit [10,11]. However, it is difficult to manually count cotton bolls because they grow in high densities. On the other hand, cotton blooms typically determine the number of cotton bolls much earlier and do not grow as densely as bolls [12]. The cotton blooms each only last a few days and grow approximately 1 to 2 months prior to harvesting. Thus, counting their frequency manually every day is also very difficult, especially for large farms [13]. Several studies have addressed these challenges in detecting and counting cotton bolls and blooms to predict yield using traditional pixel-based methods, deep learning models, and unmanned vehicles [13,14,15,16,17]. These methods use either aerial or ground vehicles that are typically equipped with cameras, on-board processors, and navigation systems to collect vast amounts of cotton image data. These data must be systematically and autonomously processed to provide for cotton boll and bloom counts, yield estimation, and potential early harvesting indicators. However, these image processing methods are linear and cannot adequately process the collected cotton image data in a timely manner. Thus, it is imperative to develop a big data architecture to autonomously process vast amounts of cotton image data in parallel. Furthermore, parallel distributed processing of large cotton datasets will provide faster and more comprehensive insights into bloom and boll development for yield estimation, harvesting timelines, and decision making.

The contribution of our research lies in the development of a low-cost, open-source, scalable, distributed computing pipeline to process vast amounts of cotton image data in parallel using Raspberry Pi, Apache Hadoop, and deep learning. Specifically, we use Hadoop to create a primary-replica distributed computing architecture to ingest batches of cotton image data, preprocess them, detect and count the number of blooms per image using our pre-trained Tiny-YOLOv4 model, and create a spatio-temporal map for each data collection day in parallel and in a distributed fashion. Moreover, we create spatio-temporal maps of cotton blooms’ appearance over time for a plot across several weeks of the growing season. These maps provide a visual understanding of the spatial distribution of bloom appearance prior to harvesting. This way, farmers have insight about the quantity of blooms that grow at what frequency over time for an entire field throughout the season. This work builds on our previous work [13], in which Tiny-YOLOv4 was used for linear spatio-temporal mapping of cotton blooms in the 2021 growing season.

The remaining sections of this paper are as follows: Section 2 provides a review of related studies using big data tools for agriculture. Section 3 details our methodology to create a distributed computing architecture for parallel processing. Section 4 presents our experimental results, and Section 5 presents the discussion and future directions. Finally, Section 6 concludes our efforts presented in this paper.

## 2. Related Works

Agricultural farms and indoor controlled environments are often equipped with several sensors, cameras, and ground or aerial vehicles that simultaneously collect data. The data collected are typically stored and processed linearly and offline [10]. However, the data that are collected over a long period of time are difficult to process and manage due to their size. Systematic handling and processing of massive amounts of data using traditional computational methods is difficult and has high latency. Incorporating big data tools has shown potential in the systematic management of large amounts of data collected. Several big data tools exist, including open-source and payment-based services. For example, open-source big data frameworks like HBase or Cassandra support tabular data and have been used in querying and numerical analysis [18,19]. Furthermore, other cloud-based services, such as Azure (see [20]), Amazon Web Services (AWS), and Teradata, are able to provide similarly robust capabilities for processing vast amounts of data, but require users to pay for the service. These cloud-based tools may become very costly for processing vast image datasets and, hence, are inapt for deployment on agricultural farms.

A few studies have used open-source, big data tools for crop monitoring. For example, the authors in [21] used Apache Spark for distributed, parallel analysis of soil quality. Specifically, the authors used soil sensors to measure soil density and compaction and developed a parallel algorithm to perform risk computations for overall plant health based on these parameters. Also, the authors in [22] used Databricks to predict soil drought based on soil sensor measurements of moisture content and temperature.

Apache Hadoop is an open-source framework that handles the processing of big data in a distributed, primary-replica architecture [23]. Hadoop allows for robust parallel processing using commodity hardware and is highly scalable to thousands of machines. Furthermore, Hadoop only requires machines to run any flavor of Linux that has Java installed, making Hadoop easily accessible from simple, single-board machines up to powerful high-computing servers. Additionally, Hadoop is fault-tolerant, provides data locality for local computation and storage, and includes data replication and compression. Several studies have used the Hadoop ecosystem for agricultural applications. For example, the authors in [24] developed an Internet of Things (IoT) alert system for irrigation using Hadoop and soil sensor data. Their system used the k-nearest neighbor algorithm along with neural networks for classifying the soil sensor data into different levels of irrigation alerts. Also, the authors in [25] used Hadoop, MapReduce, and Spark to process cabbage data using linear regression. They performed an analysis comparing the size of their virtual machine (VM) clusters and the size of their input data. Furthermore, the authors in [26] used Hadoop and Hive for crop disease analysis and solution recommendations. Their work mainly used numerical data sourced from public datasets. Lastly, the authors in [27] used Hadoop and MapReduce for crop yield prediction and incorporated the random forests algorithm based on soil data.

While the aforementioned studies show potential in the use of big data tools in agriculture, they have not shown applicability to other crops or to a variety of data or scalability to a full framework that can be used on a farm. As such, it is imperative to develop an open-source, big data pipeline that is general to a variety of crops and data, can be built and layered with other frameworks, and is scalable in terms of size. Hadoop supports a variety of data modalities, including text files, images, and even video, which contrasts with other open-source big data frameworks. This allows Hadoop to be well-suited to the variety of data that may be collected from farms or controlled environments. Thus, we selected Hadoop as our distributed computing framework in this paper.

## 3. Methodology

In this section, we describe the data collection means as well as details about the distributed big data pipeline and its deployment.

### 3.1. Cotton Farm Details

**Figure 1:**
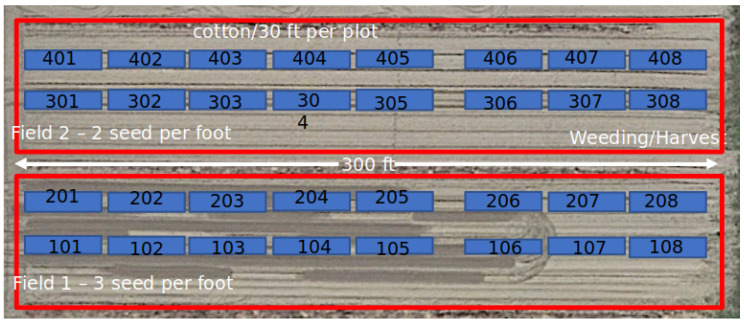
Top-down view of cotton field plot layout in Tifton, GA.

The farm we used for data collection consisted of 32 plots of cotton plants. The width of the farm was 300 feet, and each plot was 30 feet long, partitioned into 4 rows with 8 numbered plots per row. Furthermore, the farm was partitioned by different growing conditions. Namely, plots 101 through 208, referred to as ‘Field 1’, had 3 seeds per foot planted. Contrastingly, plots 301 through 408, referred to as ‘Field 2’, had 2 seeds per foot planted. The ground vehicle we used for data collection traveled up and down each plot to capture both forward and backward views of the cotton plants. An aerial view of the farm and the plot layout is shown in Figure 1.

### 3.2. Cotton Data Collection

The ground vehicle used for cotton image data collection was a four-wheel Ackermann-steer rover (Rabbit Tractors, Inc., Cedar Lake, IN, USA) from the University of Georgia in Tifton, GA. The motors were 250W Pride Mobility (Pride Mobility, Duryea, PA, USA) wheelchair motors, and the rear wheels were connected to quadrature rotary encoders, namely CUI AMT 102 (CUI, Lake Oswego, OR, USA), that provided feedback on the distance traveled by the rover. Additionally, the motors used two Cytron MDDS30 (Cytron Technologies, Pulau Pinang, Malaysia) motor controllers and were powered by two 20Ah 6-cell Turnigy LIPO batteries (Turnigy, Kwun Tong, Honk Kong, China). The rover was steered by a HDA8-50 linear servo from ServoCity (Winfield, KS, USA). These components were controlled by an Arduino Mega microcontroller (Arduino, Somerville, MA, USA). The rover was manually controlled using an IRIS+ RC transmitter from 3D Robotics (Berkeley, CA, USA), which communicated with an FRSky X8R (FrSky Electronic Co. Ltd, Brea, CA, USA) receiver connected to the Arduino.

**Figure 2:**
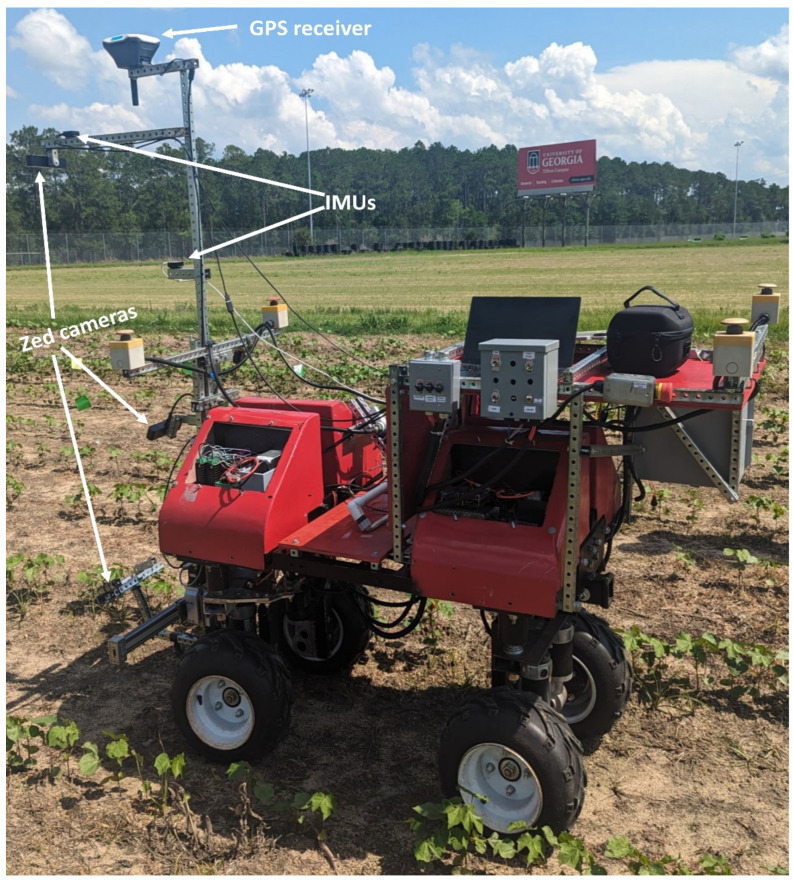
Front view of the rover deployed to collect video streams of cotton plants in Tifton, GA.

An Nvidia Jetson Xavier AGX (Nvidia Corporation, Santa Clara, CA, USA) was the embedded computer that controls all of the data collection and autonomous operations. The embedded computer was equipped with an 8-core ARM v8.2 64-bit CPU, 32GB of RAM, and a 512-core Volta GPU with tensor cores. Communication between the embedded computer and the sensors on the rover was performed using a Robotic Operating System (ROS 1 Melodic). The embedded computer was also powered by a 22Ah 6-cell Turnigy LIPO battery (Turnigy, Kwun Tong, Honk Kong, China). The rover also used a single-band EMLID Reach RS+ RTK GNSS receiver (Emlid, Budapest, Hungary), StereoLabs ZED cameras (StereoLabs, San Francisco, CA, USA) for image data collection and navigation, and two PhidgetSpatial Precision 3/3/3 High Resolution IMUs (Phidgets Inc, Calgary, AB, Canada) for tracking rover orientation and heading. A front view of the ground vehicle, electronics, and camera is shown in Figure 2.

The data was collected weekly from July 1 to October 21. Throughout the season, there were 17 data collection days. In particular, the ground vehicle collected video streams of both above- and below-canopy views of the cotton plants using two ZED cameras, ZED2 and ZEDM, respectively. In this work, we used the image data collected from the ZED2 camera to build our spatio-temporal maps, as this view provided clear views of bloom appearance throughout the growing season. Both ZED cameras have two lenses to concurrently capture both left and right views of the field data.

**Figure 3:**
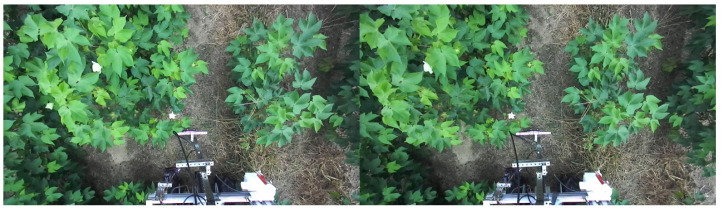
An example of ZED2 stereo camera cotton image data collected on 26 August from our research cotton farm in Tifton, GA. The image frame contains both left and right views, and several open blooms are apparent.

Additionally, as the height of the cotton plant increased throughout the growing season, the camera height was also modified accordingly. In particular, the ZED2 camera was placed 1.5 m from the ground for the first data collection day on July 1. Between 9 July and 26 August, the ZED2 camera was placed 1.64 m from the ground. Lastly, from 31 August to 21 October, the ZED2 camera was placed 1.79 m from the ground. The rover was driven down each plot and collected video stream data of the cotton plants, which were chronologically stored as ROS bag files, along with the plot number and time. The images were extracted from the bag files using rospy and CvBridge Python packages, contained within ROS (ROS 1 Melodic). An example of the extracted data from 26 August is seen in Figure 3 below.

### 3.3. Proposed Architecture Using Hadoop

**Figure 4:**
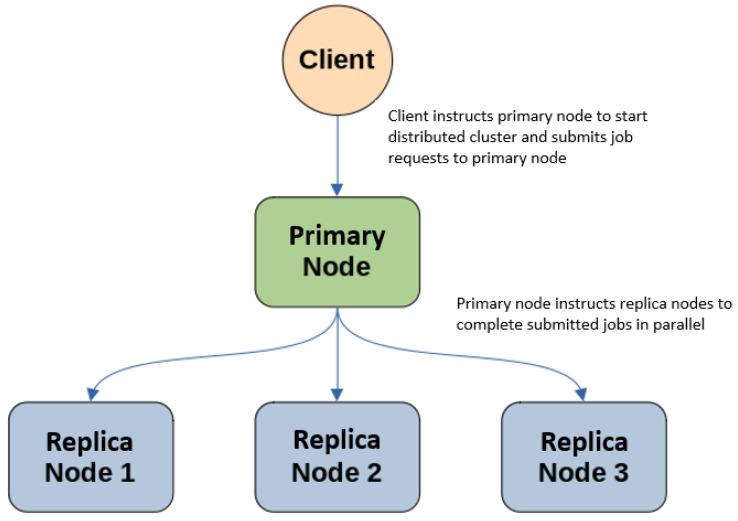
Our proposed Hadoop cluster consisting of a client, a primary node, and three replica nodes.

This section provides a brief introduction to Hadoop and the pieces used in building our distributed computing pipeline. Typical Hadoop clusters consist of a primary node, which leads the cluster, and at least one replica node, which completes jobs submitted to and assigned by the primary. These nodes can be physical or virtual machines, where nodes communicate with each other across the network. An example of our proposed distributed cluster is seen in Figure 4, with a single client, a primary node, and three replica nodes. The client starts the Hadoop cluster via the primary node and submits job requests for the replica nodes to process in parallel.

Furthermore, Hadoop contains four main components that work together: (1) Hadoop Common, which contains the core files to run Hadoop, (2) the Hadoop Distributed File System (HDFS), which serves as the big data storage component for a cluster, (3) YARN, which serves as the resource manager and job scheduler for Hadoop, and (4) MapReduce, a programming model for distributed, parallel processing of data.

**Figure 5:**
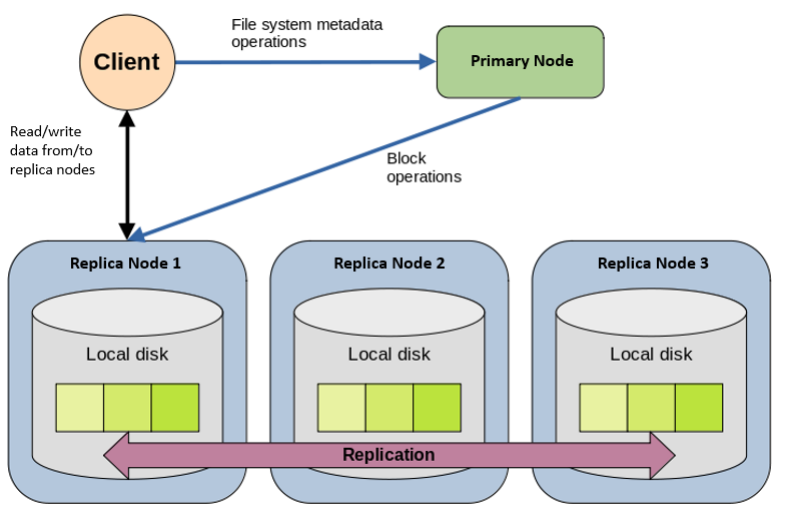
HDFS architecture for our proposed four-node cluster.

Firstly, HDFS is a distributed file system that stores data on commodity machines while being fault-tolerant and able to run on low-cost hardware. HDFS also contains a primary-replica architecture, where there is a single primary node and several replica nodes. The primary node manages the file system and access by the clients. The replica nodes manage the storage of the files in the file system. Specifically, HDFS is designed to store large files across a Hadoop cluster reliably. These large files are stored as a sequence of blocks and replicated, where each block is of a specified size of storage. The size of each block is configurable for a specified cluster and is 128 MB by default. Figure 5 shows how the client, primary node, and replica nodes are related to one another in our cluster, inspired by the original HDFS documentation in [23] and the work reported in [28].

**Figure 6:**
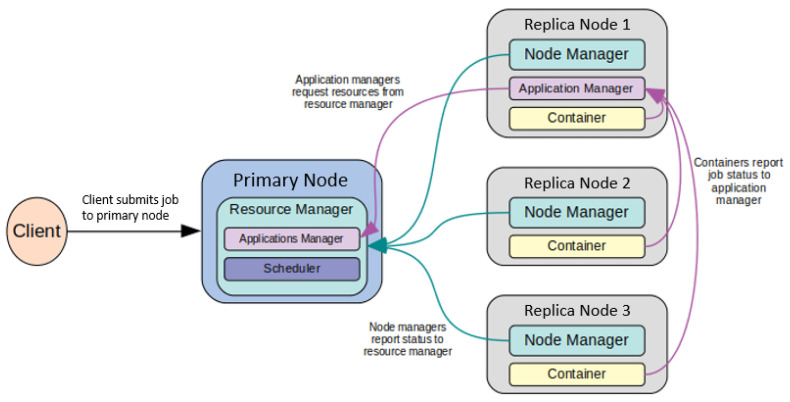
YARN architecture for our proposed four-node cluster.

Additionally, Yet Another Resource Negotiator (YARN) serves as the resource manager for a Hadoop cluster. Specifically, YARN is responsible for managing the computational resources in the cluster, scheduling them for jobs submitted by the client, and monitoring the submitted job status. Within YARN, there are several daemons that work together. In particular, the ResourceManager daemon runs on the primary node and is responsible for arbitrating computational resources for each job. Within the ResourceManager, there are two components: the Scheduler and ApplicationsManager. The Scheduler allocates computational resources to the jobs that are running and their resource requirements. The ApplicationsManager maintains the role of accepting new job submissions, negotiates resources from the Scheduler and tracks the job status. Furthermore, the NodeManager daemon runs on each replica node in the cluster and is responsible for monitoring and reporting resource usage to the ResourceManager. Figure 6 shows how the client, primary node, and replica nodes are related to one another in our cluster in terms of YARN, inspired by the original YARN documentation [23].

Lastly, MapReduce is a framework for writing jobs that enable a Hadoop cluster to reliably process large amounts of data in parallel, even on commodity hardware. These jobs split the input data from HDFS into smaller partitions and are distributed across the cluster. This is referred to as the map task. The smaller partitions are then processed as per the job configuration, and outputs are sorted and stored together in a single folder in HDFS. The sorting of outputs after the map tasks and processing are referred to as the reduce tasks. The benefit of the MapReduce framework is that it also handles the scheduling of jobs, monitoring of jobs, and re-executing of failed jobs. We wrote our MapReduce jobs in Java using the Writeable Interface. The codes for image splitting were inspired by this GitHub repository, accessed on 1 September 2022, (https://github.com/sozykin/mipr) and were compiled using Maven version 3.6.3.

### 3.4. Development of Proposed Hadoop Distributed Computing Cluster

**Figure 7:**
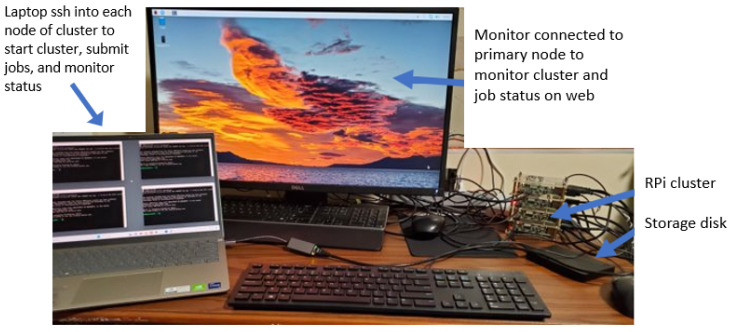
An overview of our distributed computing cluster setup with four nodes.

**Figure 8:**
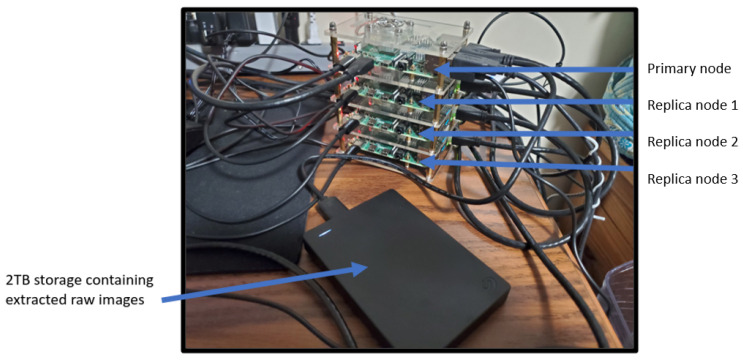
A closeup of our distributed computing cluster setup with four nodes.

Our distributed cluster consists of a single primary node and three replica nodes, where each node is a Raspberry Pi Model 4B, each with 4 GB of RAM, 32 GB SD card, and the official 32-bit Raspbian OS. The extracted raw cotton image data are stored on a 2 TB external hard disk from SeaGate (Dublin, Ireland). The external hard disk is connected to the primary node via its on-board USB 3.0 port. Also, each node has its own designated power supply, and the nodes are connected together using an unnetworked Ethernet hub. One port of the Ethernet hub is connected to the client laptop, which uses PuTTy to remotely ssh into each node of the cluster. The client laptop was a Dell Inspiron 14 5420 with Windows 11 OS, Intel Core i7-1255U, 16 GB DDR4 RAM, 512 GB SSD, and an NVIDIA GeForce MX570 GPU (Dell, Round Rock, TX, USA). The client starts the cluster via the primary node and monitors the status of the replica nodes using the Hadoop web interface and the remote ssh PuTTy terminals. The nodes are stacked together using a cluster casing from GeeekPi (Shanghai, China), with a small fan above each node’s CPU, and CPU heatsinks to minimize thermal thresholding. The physical setup is shown in Figure 7. Also, a closeup view of the cluster and storage unit is shown in Figure 8.

Each node used Hadoop version 3.3.4 from the official Hadoop mirrors. Hadoop only requires that each node use a flavor of GNU Linux, Java 8, and Secure Shell ssh. Once Hadoop and its necessary packages are installed, each node is configured based on its role, either primary or replica, and connected together via the Ethernet hub. Each node has a unique IP address and can effectively communicate with other nodes. Furthermore, a common user between the nodes is set to ensure effective cluster usage. Typically, this means that each node has the same username but different hostnames and IP addresses to differentiate one from another. Moreover, the primary node maintains a list of the replica nodes. For the primary node to have access to the HDFS for storage, the primary node also acts as a DataNode to process data in parallel with the replica nodes. Thus, the primary node does not remain idle after receiving job requests from the client. The client starts the cluster via the primary node by formatting and starting HDFS. The client then creates the necessary directories that allow MapReduce jobs to execute and loads batches of cotton image data into the HDFS organized by date and row number. The client starts YARN for resource management and executes image processing jobs for image splitting and spatio-temporal map creation in parallel. Additionally, each node processes each split image for cotton bloom detection in parallel.

### 3.5. Image Preprocessing on Clusters Using MapReduce

The first step in preprocessing the cotton image data using our cluster is data extraction and image splitting. For each row in our cotton farm, the images were extracted from the bag files and stored on the external hard disk. Once the cluster was started via client instruction on the primary node, the raw cotton image data were inputted into HDFS. The images were stored by date and the plot number that they corresponded to. Next, we created a MapReduce job to split the ZED2 camera image data in half to separate the views from both the left and right lenses. In particular, the input to our MapReduce job was the HDFS folder containing the cotton image data. Each image within the HDFS folder was processed by reading the image as a BufferredImage, splitting the image in half to separate the left- and right-hand lenses, and storing both split images into a new HDFS folder as the output.

**Figure 9:**
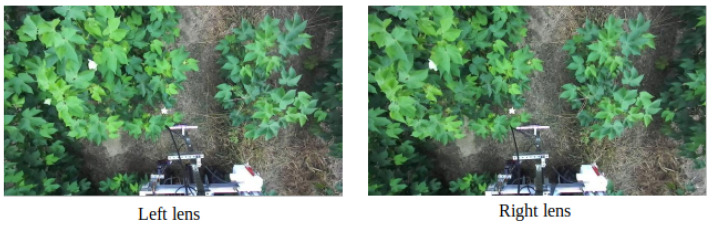
Figure 3 split into the left and right image frames using MapReduce and our distributed cluster.

Once we start the clustering and loading the data into HDFS, we start the resource manager, YARN, via the primary node. After YARN is started, our primary node is ready to receive job requests from the client. We submit separate job requests for each plot of data for image splitting and store the split output in new folders corresponding to the processed plot. We also record the processing time for image splitting on the cluster. An example of the image splitting job output is shown in Figure 9, which is Figure 3 split into the left and right image frames.

### 3.6. Cotton Bloom Detection Using Tiny-YOLOv4 and MapReduce

After we split the images and store the split images as output into the HDFS, we process these images by performing cotton bloom detection using Tiny-YOLOv4 and MapReduce. Specifically, we use the cotton bloom detection model from our previous work in [13]. In particular, the Tiny-YOLOv4 model uses fewer layers than the official YOLO model and performs object detection and localization in a single pass. Also, the Tiny-YOLOv4 model differs from its prior generations in that it uses mosaic data augmentation to increase training accuracy and model performance with smaller datasets [29,30]. Our model was trained from scratch using 2000 annotated images of cotton blooms from the 2021 growing season collected from the same farm in Tifton, GA. The model was trained using Darknet and had a high F1 Score of above 0.95, a true positive rate of 98%, and low false negative and false positive rates.

The inputs for our bloom detection job are the split images stored in the HDFS. Our MapReduce job for distributed bloom detection uses the OpenCV Deep Neural Network (DNN) module to read and load our pre-trained Tiny-YOLO model. The DNN module has specific functions for reading and loading YOLO models directly from Darknet configuration and weight files. The images are processed for bloom detection for each plot’s split images, and the output image is stored on the HDFS. Additionally, we store the detected bloom coordinates locally on the primary node. Specifically, for each image that contains at least one detected cotton bloom, a corresponding text file is populated with the bounding box coordinates of all the detected blooms within that image. These text files are shared among all the nodes in the cluster for spatio-temporal map creation using MapReduce. Given that our cluster has already been started by the client, the data have been loaded into HDFS, and YARN has started, we submit separate job requests to the primary node for cotton bloom detection and store the coordinate text files in the cluster corresponding to the processed plot. We also record the processing time for cotton bloom detection on the cluster.

### 3.7. Spatio-Temporal Map Creation Using MapReduce

Following distributed bloom detection, we create spatio-temporal maps to visualize the spatial distribution of cotton bloom appearance in the same area over time. We create a MapReduce job to build the spatio-temporal maps. Specifically, the input to the job is the HDFS folders containing the split images for the same plot over time. Then, the job searches for the input image’s corresponding text file containing the coordinates of detected blooms stored locally on the replica nodes, reads and parses the coordinates, and draws the colored boxes on the image at the parsed coordinates in a color corresponding to the plot’s date. The output, which is stored in the HDFS, is images with colored boxes corresponding to detected blooms at the current time point. The job is re-executed for a later date from the same plot. The final output is the spatio-temporal map with colored boxes corresponding to detected blooms at different locations and instances of time in the same plot. From this final output, we examine the spatial distribution of bloom appearance over time on the same plot.

### 3.8. Evaluation of Distributed Cluster and Model

To evaluate the performance of our developed pipeline, we compare the accuracy of our pre-trained Tiny-YOLOv4 model with the new season’s training data, analyze the average processing time of the cluster, and analyze the total CPU usage of each of the nodes. Since we are interested in similarly creating spatio-temporal maps of cotton bloom appearance, we tested the performance of our pre-trained Tiny-YOLOv4 model on cotton image data from the 2022 growing season. If using the Tiny-YOLOv4 model on the cotton image data from 2022 leads to high performance in detecting cotton blooms from the 2021 growing season, then we are able to use the model for our cluster to create spatio-temporal maps. In particular, we compare the model’s performance in terms of its difference in count (DiC), its mean difference in count (|DiC|), mean squared error (MSE), F1 Score, true positive rate (TPR), false positive rate (FPR), and false negative rate (FNR) on the 2022 growing season with its original performance on the 2021 growing season. Ideally, the metrics for DiC, |DiC|, and MSE should be low and near 0. The metrics for the F1 Score should be high and near 1.0. Lastly, the metrics for TPR should approach 100%, while the FPR and FNR should both be low and near 0%.

In addition to presenting the processing time of the cluster, we also evaluate the CPU usage of the cluster while the cluster is idle and when the cluster is running a job. Ideally, a distributed cluster should use nearly 100% of all of the CPU cores when running a job for each node. This way, the cluster is making full use of the computational power available to process the data. When the cluster is idle, the CPU usage should ideally be low, less than 10% per node. Finally, we present the cost of our cluster, indicating that it is a low-cost, scalable, and effective distributed computing cluster.

### 3.9. Summary of Methodology

**Figure 10:**
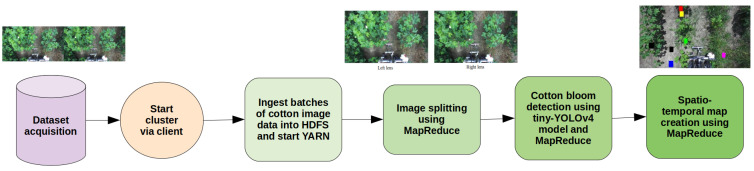
Summary of our proposed workflow in this paper.

In Figure 10, we portray our pipeline, including data acquisition and cluster start as well as distributed image preprocessing, bloom detection, and final spatio-temporal map creation. The codes used in these experiments will be made available on our lab website.

## 4. Results

First, we test the performance of our pre-trained Tiny-YOLOv4 model on 100 split images from different rows from August 22 and August 26, as most blooms appeared during these days and the plants had not yet begun to defoliate. We compare the same metrics discussed in [13], particularly, the difference in count (DiC), absolute DiC, mean squared error (MSE), F1 Score, true positive rate (TPR), false positive rate (FPR), and false negative rate (FNR). Table 1 summarizes the comparison of the performance of the pre-trained Tiny-YOLOv4 model on our 2022 testing set and the same testing set as in [13].

It is notable that the average MSE and F1 Score decreased, but the TPR remains at least 93%. The lower MSE and F1 Score are attributed to the higher FPR as the model incorrectly detects parts of the rover or background shapes that are similar to blooms as cotton blooms. Since the overall performance of the Tiny-YOLOv4 is high and the model can still accurately detect blooms even with the new datasets, we proceeded to incorporate the Tiny-YOLOv4 model into our cluster. The model is deployed onto each of the four nodes in the cluster, and a MapReduce job loads the model weights and performs cotton bloom detection in a distributed fashion.

Next, we evaluate the performance of our cluster in terms of processing time on a plot of data that contained 123 images. These image data were stored on the HDFS and used for distributed and parallel processing by our cluster. As per our methodology, the input data are split, fed into our pre-trained Tiny-YOLOv4 model for distributed bloom detection, and finally postprocessed to create the spatio-temporal maps. Table 2 summarizes the processing time of our distributed cluster for the main three jobs: image splitting, cotton bloom detection, and spatio-temporal map creation.

We also compare our cluster performance on the same 123 images in one-node, two-node, and three-node clusters. The purpose of this comparison is to determine if having more nodes will affect the processing time of the cluster when running jobs, as the resource manager must spend more computational overhead determining whether the nodes have enough resources to process the job. Additionally, a greater computational burden is placed on the primary node to manage the cluster with the addition of more nodes. Table 3 summarizes the result of the comparison. From this table, we see that the performance of higher node clusters is faster than that of smaller node clusters, as the workload and data are distributed across more replicas. Thus, having more nodes in a distributed cluster is effective in reducing the computational time spent processing the cotton image data. Additionally, this indicates that our cluster is easily scalable, and more nodes can be added to further reduce the computational processing time.

Additionally, we compare our cluster performance to that of a centralized VM. The VM is created using a 32-bit Debian 11.7 OS, 16 GB of RAM, and 1 CPU with Hadoop 3.3.4 on Oracle VirtualBox (Oracle Corporation, Austin, TX, USA). The same MapReduce jobs and OpenCV dependencies were used in this VM. The purpose of this comparison is to determine if a distributed computing cluster is faster at processing cotton image data than a linear, centralized machine. Table 4 summarizes the result of this comparison. From this table, we observe that the performance of a similarly powered, centralized VM is significantly slower than our distributed Raspberry Pi cluster. This, in turn, justifies the use of a distributed computing cluster instead of a centralized machine since the workload is shared across a cluster.

Lastly, we examine the performance of our cluster when the input size is significantly larger and whether the cluster is able to process all of the input without failure. We tested our cluster with one day worth of image data collected from the cotton field. We used the data from August 9, which contained 7235 images (2.4 GB). We loaded the data onto the HDFS and processed the images as per our methodology. Our cluster was able to successfully process the large quantity of data without failure, indicating that our cluster is robust and has sufficient resources when running jobs for long periods of time. Table 5 summarizes the performance of the cluster on one day worth of data.

Furthermore, we used the Unix command htop to analyze the CPU usage of our cluster while it remained idle and while it was running jobs. When our cluster was idle with the resource manager running, the CPU usage ranged between 1% and 6% for all four cores for each node. This indicates ideal CPU performance for a distributed cluster, both when the resource manager was running and when it remained idle. When our cluster was running jobs, the CPU usage ranged from 90% to 100% for all four cores for each node. This implies that our distributed cluster utilizes the full computational power available by the CPU cores for each node when running jobs.

To visualize the final, comprehensive spatio-temporal maps, we select nine data collection days from the same plot, namely 29 July, 4 August, 9 August, 15 August, 22 August, 26 August, 7 September, 16 September, and 23 September. Specifically, we select plot 101 from the cotton field, as described in our methodology. 29 July was selected as the base date and served as the background for our spatio-temporal maps. During this period of the growing season, the cotton plants were very small, and few blooms were apparent.

**Figure 11:**
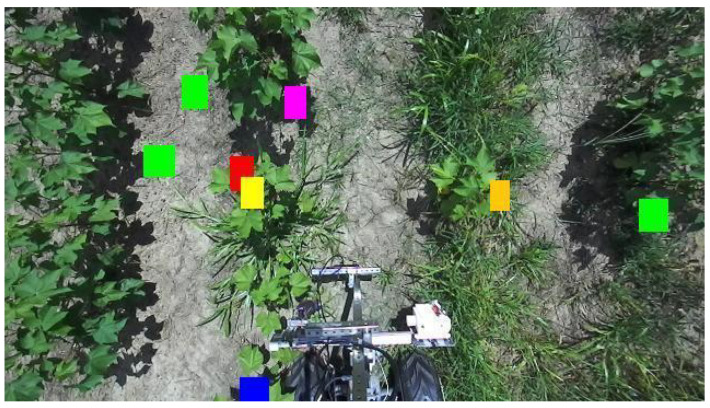
An illustrative example of our spatio-temporal maps.

The following dates in August and September are used to overlay later bloom locations onto the base date using different colored boxes for each date. 4 August corresponds to red boxes, 9 August to orange boxes, 15 August to yellow boxes, 22 August to green boxes, 26 August to blue boxes, 7 September to cyan boxes, 16 September to magenta boxes, and 23 September to black boxes. The images from these dates in August and September are fed into our cluster for image splitting and bloom detection. The saved coordinates are used to overlay bloom locations using colored boxes from the same 101 plots from later dates onto the 29 July base date. These colored boxes represent the spatial distribution of the cotton bloom appearance at later dates throughout the growing season. Additionally, the spatial distribution of the blooms on this plot from later dates can be used to estimate the rate of bloom appearance for an entire growing season. Figure 11 shows an example of our spatio-temporal maps. In this figure, we see more green colored boxes, which correspond to the later blooms appearing on 22 August at those locations, indicating that more blooms appeared during late August for this plot.

Finally, Table 6 summarizes the total cost of our cluster. From this table, we see that, for under USD 500, we were able to develop a fully open-source, distributed computing pipeline that can robustly process cotton image data. With our cluster being low-cost, its adoption is feasible for farmers.

## 5. Discussion and Future Directions

In this work, we developed a distributed computing pipeline to process cotton image data. While our work has contributed by being the first open-source architecture for cotton phenotyping applications, several lines of future work may improve the overall performance of our distributed computing architecture.

Firstly, our cluster only has four nodes, with a single primary node and three replica nodes. Hadoop is capable of running with up to thousands of machines effectively. Also, increasing the number of nodes in the cluster will further improve the computational time to process the cotton image data in parallel. To accomplish this, each of the replica nodes should have similar hardware configurations. The primary node, however, should have an increased amount of RAM to accommodate the increased number of replica nodes, as significantly more metadata about the entire cluster must be stored and resources must be managed. The addition of more nodes to our cluster would be subject to further investigation of its performance in terms of computational time and CPU usage.

Secondly, our MapReduce jobs have long processing times for large datasets. This effect would be compounded if all data input sizes were similarly large. Thus, it is imperative to reduce the computational time for running jobs on large datasets. Transitioning our MapReduce codes to Apache Spark [31] would significantly improve processing time to near real-time. Apache Spark is different from MapReduce as it uses resilient distributed dataset (RDD) abstraction. Moreover, Spark requires an extensive amount of RAM, which drastically increases the initial cost of Spark in comparison to MapReduce, as it can run on low-cost, commodity hardware, as demonstrated in this work. Spark can also be configured to function with Hadoop and HDFS, making our work easily scalable.

Thirdly, our cluster processed only image data in this paper. However, Hadoop is also capable of handling a variety of data types, including numerical, tabular, and video. Thus, capturing and processing different types of data would allow our cluster to be used for any general-purpose processing for precision farming applications. For example, our cluster may be able to process numerical sensor measurements that are collected from the field. Furthermore, processing numerical data to analyze growth trends can be performed using traditional machine learning methods and with the Machine Learning library in Apache Spark.

Lastly, our cluster processed batches of cotton image data that have already been collected, extracted, and stored onto an external disk. However, it may be more efficient to directly stream the collected data from the field onto the cluster for immediate processing. Furthermore, implementing data streaming would aid in reducing offline data storage and preprocessing before cluster usage. For example, the data that are being collected on the farm can be streamed directly to the cluster for immediate processing instead of offline or overnight processing. Apache Spark can also provide this capability.

## 6. Conclusions

In this paper, we developed a low-cost, open-source, distributed computing architecture using Hadoop and deep learning for spatio-temporal mapping of cotton bloom appearance for an entire season. In particular, our method employed a cluster of Raspberry Pis in a primary-replica architecture to ingest batches of cotton image data, preprocess them, perform cotton bloom detection, and create spatio-temporal maps in parallel. Moreover, our cluster demonstrated improved performance in terms of faster computational time in comparison to a single, centralized node with the same accuracy.

## Figures and Tables

**Table 1:** Network evaluation metrics comparing pre-trained Tiny-YOLOv4 performance on new cotton image data versus 2021 cotton image data.

Metric	2022	2021
DiC	−0.525	−0.059
|DiC|	0.71	0.119
MSE	1.09	0.119
F1 Score	0.825	0.967
TPR	93.07%	98.52%
FPR	25.26%	6.25%
FNR	6.93%	1.48%

**Table 2:** Distributed cluster performance on processing cotton image data.

Job	Total Time	Total Images	Average Time ($\frac{\sec}{\mathrm{image}}$)
Image splitting	1:59	123	0.96
Cotton bloom detection	5:32	246	1.349
Spatio-temporal map creation	1:54	246	0.48
Total time	9:25		

**Table 3:** Comparison of performance with smaller node clusters.

Job	One-Node Cluster	Two-Node Cluster	Three-Node Cluster
Image splitting	7:03	3:39	2:43
Cotton bloom detection	21:31	10:37	7:13
Spatio-temporal map creation	13:11	6:23	4:23
Total time	41:45	20:39	14:19

**Table 4:** Performance of centralized VM.

Job	Centralized Processing Time
Image splitting	8:54
Cotton bloom detection	36:54
Spatio-temporal map creation	17:32
Total time	1:03:23

**Table 5:** Cluster performance on one day worth of data.

Job	Total Processing Time
Image splitting	1:29:21
Cotton bloom detection	4:53:56
Spatio-temporal map creation	2:53:49
Total time	9:16:56

**Table 6:** Cost of our distributed cluster.

Item	Quantity	Cost per Unit
Raspberry Pi 4 (4 GB of RAM)	4	USD 74.00
Raspberry Pi cluster case	1	USD 19.99
32GB SD card (two-pack)	2	USD 8.49
Ethernet cable	5	USD 3.50
Ethernet hub	1	USD 8.99
Ethernet adapter	1	USD 13.45
USB-C power cable (five-pack)	1	USD 24.60
HDMI to micro-HDMI cable	1	USD 11.20
2TB Seagate hard disk	1	USD 67.94
Total cost		USD 476.65

## Data Availability

Data are contained within the article.

## Abbreviations

The following abbreviations are used in this manuscript:

HDFS	Hadoop Distributed File System
YARN	Yet Another Resource Negotiator
DiC	Difference in Count
|DiC|	Absolute Difference in Count
MSE	Mean square error
TPR	True positive rate
FPR	False positive rate
FNR	False negative rate
DNN	Deep Neural Network
YOLO	You Only Look Once
VM	Virtual machine
